# Strategic and Dynamic Temporal Weighting for Perceptual Decisions in Humans and Macaques

**DOI:** 10.1523/ENEURO.0169-18.2018

**Published:** 2018-10-15

**Authors:** Aaron J Levi, Jacob L. Yates, Alexander C. Huk, Leor N. Katz

**Affiliations:** 1 Department of Neuroscience, the University of Texas at Austin, Austin, Texas; 2 Department of Psychology, The University of Texas at Austin, Austin, Texas; 3 Center for Perceptual Systems, The University of Texas at Austin, Austin, Texas; 4 Center for Visual Science, University of Rochester, Rochester, New York; 5 Department of Brain and Cognitive Sciences, University of Rochester, Rochester, New York; 6 Laboratory of Sensorimotor Research, National Eye Institute, National Institutes of Health, Bethesda, Maryland

**Keywords:** Decision making, perceptual decisions, psychophysics, reverse correlation, temporal weighting

## Abstract

Perceptual decision-making is often modeled as the accumulation of sensory evidence over time. Recent studies using psychophysical reverse correlation have shown that even though the sensory evidence is stationary over time, subjects may exhibit a time-varying weighting strategy, weighting some stimulus epochs more heavily than others. While previous work has explained time-varying weighting as a consequence of static decision mechanisms (e.g., decision bound or leak), here we show that time-varying weighting can reflect strategic adaptation to stimulus statistics, and thus can readily take a number of forms. We characterized the temporal weighting strategies of humans and macaques performing a motion discrimination task in which the amount of information carried by the motion stimulus was manipulated over time. Both species could adapt their temporal weighting strategy to match the time-varying statistics of the sensory stimulus. When early stimulus epochs had higher mean motion strength than late, subjects adopted a pronounced early weighting strategy, where early information was weighted more heavily in guiding perceptual decisions. When the mean motion strength was greater in later stimulus epochs, in contrast, subjects shifted to a marked late weighting strategy. These results demonstrate that perceptual decisions involve a temporally flexible weighting process in both humans and monkeys, and introduce a paradigm with which to manipulate sensory weighting in decision-making tasks.

## Significance Statement

During decision-making, the weight assigned by subjects to sensory information over time is not necessarily constant. Such time-varying weighting is often interpreted as a signature of a particular decision-making model (e.g., higher weighting of early stimulus information is consistent with a bounded accumulation process). Temporal weighting may also result, however, from a strategic reweighting of the stimulus evidence itself that takes place before and/or independent of a decision-making mechanism. Here we use a psychophysical reverse correlation paradigm to both measure and manipulate temporal weighting behavior. We demonstrate that both humans and macaques adopt weighting strategies that are flexible, consistent with dynamic reweighing of the sensory stimulus.

## Introduction

Perceptual decisions are typically thought of as resulting from some form of accumulating samples of a stimulus over time. During this process, a decision variable is updated as evidence is integrated until a choice is made. In both human and nonhuman primates, perceptual decision-making has been studied extensively in the context of motion direction discrimination tasks, where the vast majority of stimuli provide statistically uniform sensory evidence over time (Gold and Shadlen, 2007). Despite a stationary level of expected sensory evidence, subjects often assign more weight to some stimulus epochs over others. In many instances, subjects have exhibited “early weighting,” where sensory evidence presented in early epochs contributes more to choices than that in late (Huk and Shadlen, 2005; Kiani et al., 2008, Nienborg and Cumming, 2009; Yates et al., 2017). In other instances, however, “late weighting” has been observed, where choices were primarily influenced by sensory evidence presented in late stimulus epochs (Tsetsos et al., 2012; Cheadle et al., 2014; Bronfman et al., 2016; Carland et al., 2016). In rodents, a mixture of either early or flat weighting profiles has been reported (Erlich et al., 2015; Scott et al., 2015; Pinto et al., 2017; Licata et al., 2017).

The diverse set of temporal weighting profiles observed across studies and species may be explained in a number of ways. One approach appeals to mechanistic models of decision-making. An early weighting strategy, for example, could be explained as a consequence of bounded accumulation (Huk and Shadlen, 2005; Kiani et al., 2008), which posits that sensory evidence is accumulated until reaching a bound, whereupon the decision is made. Because the remainder of the stimulus is ignored once the bound has been hit, early stimulus epochs contribute more to decisions than late. Late weighting, in contrast, may be interpreted as a consequence of leaky accumulation (Usher and McClelland, 2001), which stipulates that the representation of sensory evidence decays over time. In this model, early sensory evidence contributes less to decisions compared to late.

An alternative approach to explaining the variety in weighing strategies postulates that the temporal weighting strategy is flexible and is linked to the demands or structure of the task. This notion is supported by experiments in which weighting changes systematically with variable trial length and signal timings (Ghose 2006; Tsetsos et al., 2012; Ossmy et al., 2013; Bronfman et al., 2016), as well as by studies that explore effects of congruency between serially presented samples (Cheadle et al., 2014). Irrespective of a stipulated model or mechanism, these studies point to similar conclusions: subjects may reweigh stimulus information as dictated by the reliability of the evidence and demands of the task.

Without appeal to a specific decision-making mechanism, we set out to manipulate temporal weighting under the hypothesis that weights should be flexible and influenced by the dynamic features of the stimulus itself, either independent of or in addition to constraints imposed by integration mechanisms such as a bound or a leak.

**Figure 1.**
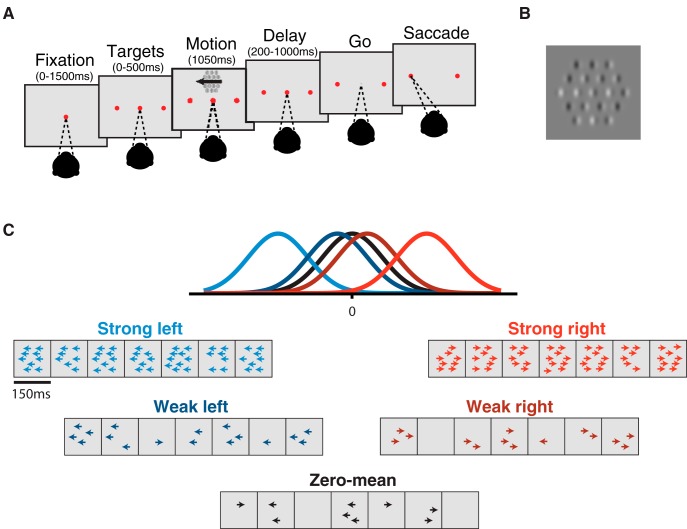
Sequence of trial events. ***A***, Subjects fixated on a central point through the appearance of targets and motion stimulus until the disappearance of the fixation point (“go”). Choices were made with saccades to the target corresponding to the perceived net direction of motion. Initial fixation time, target-on duration, and time until fixation point disappearance were randomly varied. ***B***, An example frame of the Gabor motion pulse stimulus. The stimulus is composed of 19 Gabor patches, where motion strength is denoted by the proportion of coherently drifting Gabors out of the total number elements in the stimulus. ***C***, Motion pulse values are generated from Gaussian distributions spanning a large range of possible motion strengths in either direction. A single trial consists of seven motion pulses, each randomly drawn from one of the Gaussians. Example trials with pulses drawn from each Gaussian (strong left/right, weak left/right, and zero-mean) are presented in cartoon form where the number of arrows represents the number of coherently drifting Gabor elements.

**Figure 2.**
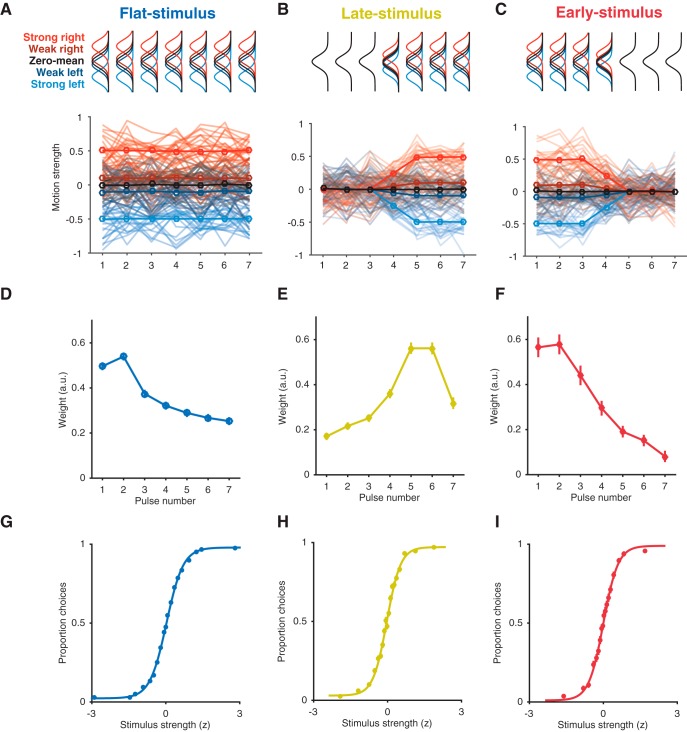
Temporal weighting profiles and psychometric functions for humans and macaques across flat-, late-, and early-stimulus conditions. ***A–C***, Top: schematic of the Gaussian distributions that generate the motion pulses. In the flat-stimulus (***A***), Gaussians remain stationary over time. In the late-stimulus (***B***) and early-stimulus (***C***) conditions, the distribution means for signal trials are varied over time. Bottom: example sessions for each stimulus condition. Motion pulse values are drawn from their color-matched Gaussians on each pulse such that the mean of many trials (bold line) reflects the temporal structure of the mean of the Gaussians. Motion pulse values in individual trials (semitransparent traces) vary considerably, in accordance with the variance of color-matched Gaussians. ***D–F***, Temporal weighting profiles averaged across all subjects (human and macaque) and sessions within the flat-stimulus (***D***), late-stimulus (***E***), and early-stimulus (***F***) conditions, showing the mean weight assigned to each of the seven motion pulses. Error bars represent ± 1 SEM. ***G–I***, Psychometric performance averaged over all sessions for flat-stimulus (***G***), late-stimulus (***H***), and early-stimulus (***I***) conditions, fitted by a logistic function capturing the dependence of choice on stimulus strength. Error bars represent ± 1 SEM (often occluded by points).

To test this idea, we adopted a motion stimulus designed explicitly for psychophysical reverse correlation in the presence of experimenter-controlled manipulation of temporal stimulus statistics (Katz et al., 2016, Yates et al., 2017). The stimulus is similar to classic motion stimuli used in the study of perceptual decisions (Newsome and Paré, 1988; Britten et al., 1992), but with two crucial features: (a) the stimulus consists of seven consecutive motion pulses, each with a predetermined mean motion strength and direction, and thus can be precisely designed to carry more or less motion evidence at different epochs (Fig. 1); (b) the stimulus is amenable to psychophysical reverse correlation analysis such that subject temporal weighting strategy may be computed directly. This motion discrimination task was performed under three temporal conditions: (1) “flat-stimulus,” in which the mean motion strength per pulse was constant; (2) “early-stimulus,” in which early pulses had high mean motion strength and late pulses had low; and (3) “late-stimulus,” in which late pulses had high mean motion strength and early pulses had low (Fig. 2*A–C*). In all conditions, the task was to report the net motion of the trial.

We found that in both time-varied conditions (early-stimulus and late-stimulus), subjects shifted their temporal weighting strategy, placing highest weight on motion pulses with the highest mean motion strength. In flat-stimulus sessions, however, subjects exhibited a large range of temporal weighting strategies despite equal mean motion strength over time. Overall, these results demonstrate that temporal weighting strategies in human and monkey observers are flexible and can be adjusted to suit temporal stimulus statistics.

## Materials and Methods

### Subjects and apparatus

Data were collected from both monkeys and humans. Monkey data were collected from two adult rhesus macaques (one female and one male, referred to as M1 and M2 hereafter) aged 10 and 14 years, weighing 7.7 and 10 kg, respectively. All animal procedures were performed in accordance with The University of Texas at Austin animal care committee’s regulations. Both M1 and M2 had standard surgery for implantation of a head-holder. Some portion of the monkey data were presented previously (Katz et al., 2016; Yates et al., 2017). Human data were collected from three subjects (all males, referred to as H1, H2, and H3), aged 23–41 years, all with normal or corrected-to-normal vision. Experiments were performed with the written consent of each observer and all procedures were approved by The University of Texas at Austin review board.

For both monkeys and humans, stimuli were presented using the Psychophysics Toolbox with Matlab (Mathworks) using a Datapixx I/O box (Vpixx) for precise temporal registration (Eastman and Huk, 2012). Sample stimulus presentation code is available on request. Eye position was tracked using an Eyelink eye tracker (SR Research), sampled at 1 kHz. Monkeys sat in a primate chair (Crist Instruments) and viewed stimuli on a 55-inch LCD (LG) display (resolution = 1920 × 1080p, refresh rate = 60 Hz, background luminance = 26.49 cd/m^2^) that was corrected to have a linear gamma function. Monkeys viewed the stimulus from a distance of 118 cm (such that the screen width subtended 54 degrees of visual angle, and each pixel subtended 0.0282 degrees of visual angle). Auditory feedback was played at the end of every trial, and fluid reward was delivered through a computer-controlled solenoid. Humans viewed stimuli on a linearized 16.5-inch OLED (LG) display (resolution = 1920 × 1080p, refresh rate = 60 Hz, background luminance = 67.22 cd/m^2^) at a distance of 65.3 cm (such that screen width subtended 31 degrees of visual angle, and each pixel subtended 0.0163 degrees of visual angle).

### Task and stimulus design

Stimulus and task design were identical between monkeys and humans unless otherwise noted. Subjects were required to discriminate the net direction of a motion stimulus and communicate their decision with an eye movement to one of two targets, placed on either side of the motion stimulus. The sequence of task events is presented in Fig. 1. A trial began with the appearance of a fixation point. Once the subject acquired fixation and held for 400–1200 ms (uniform distribution), two targets appeared and remained visible until the end of the trial. 200–1000 ms after target onset, the motion stimulus was presented at a range of eccentricities from 4° to 10° for a duration of 1050 ms. The fixation point was extinguished 200-1000 ms after motion offset, and the subject was then required to shift their gaze toward one of the two targets within 600 ms (saccade end points within 3° of the target location were accepted). The timing of each event was randomly and independently jittered from trial to trial (Fig. 1*A*).

The reverse-correlation motion stimulus contained motion toward one direction or the opposite, with varying motion strength. Spatially, the stimulus consisted of a hexagonal grid of 19 Gabor elements, 5°–7° across, scaled by eccentricity (Fig. 1*B*). Individual Gabor elements were set to approximate the receptive field (RF) size of a V1 neuron, and the entire motion stimulus approximated the RF size of an MT neuron (Van Essen et al., 1981). Motion was presented by varying the phase of the sine-wave carrier of the Gabors. Each Gabor underwent a sinusoidal contrast modulation over time with independent random phase to prevent perceptual “pop-out” of individual drifting elements. Gabor spatial frequency (0.8 cycles/°, sigma = 0.1 × eccentricity) and temporal frequency 5–7 Hz, yielding velocities of 5.55–7.77°/s, respectively) were selected to match the approximate sensitivity of MT neurons (Bair and Movshon, 2004).

Each motion stimulus presentation consisted of seven consecutive motion pulses lasting 150 ms each (9 frames), producing a motion sequence of 1050 ms in duration in total. For human subjects S2 and S3, each motion pulse lasted 100 ms each (6 frames), producing a 700-ms-long stimulus. On any given pulse, a number of Gabor elements would have their carrier sine waves drift in unison to produce motion (“signal elements”), and the remaining would counterphase flicker (“noise elements”). Signal elements on any given pulse were assigned at random within the grid and all signal element drifted in the same direction. Motion strength on pulse *i* was defined as the proportion of signal elements out of the total number of elements, the value of which was drawn from a Gaussian distribution, $X_{i}∼N\left(\mu_{k},\sigma \right)$ and rounded to the nearest integer, where *k* is the distribution index for the five trial types (strong left, weak left, zero-mean, weak right, strong right) and $\mu_{k}$ was one of five values: –50%, –10%, 0%, 10%, and 50% (sign indicates motion in the opposite direction), and σ was set to 15%. Thus, while each pulse within a sequence could take on any value (and either sign/direction) from distribution $N\left(\mu_{k},\sigma \right)$, the expectation of a sequence would be $\mu_{k}$ (Fig. 1). The subjects were rewarded for selecting the target consistent with the sign of the motion pulse sequence sum (i.e., the net direction), independent of the distribution $\mu_{k}$ from which the pulses were drawn.

The distributions $N\left(\mu_{k},\sigma \right)$ were most commonly set to the values listed above but were occasionally varied to better maintain individual subject performance around threshold. Overall, humans performed sessions with $\mu_{strong}$ ranging from 35% to 50% and $\mu_{weak}$ ranging from 10% to 20%, with σ ranging from 10% to 24% coherence. Macaques performed sessions with $\mu_{strong}$ ranging from 50% to 70% and $\mu_{weak}$ ranging from 10% to 20%, with σ ranging from 8% to 24% coherence.

### Temporal manipulation of stimulus

In the standard stimulus design described above, the mean of the motion strength distribution $N\left(\mu_{k},\sigma \right)$ would be held constant throughout a stimulus presentation. In other words, the mean of the distribution from which $X_{i}$ was drawn was fixed at $\mu_{k}$, for pulses 1–7 (Fig. 2*A*). We refer to this as the “flat-stimulus” condition and treat it as a baseline, because it is similar to most variants of the classic moving dot stimuli used in the past (Newsome and Paré, 1988; Britten et al., 1992, 1996; Gold and Shadlen 2007). In the time-varying stimulus conditions (the early-stimulus or late-stimulus), $\mu_{k}$ was varied over pulses 1–7. Fig. 2*B* depicts a stimulus condition in which motion strength is reduced substantially in early pulses (relative to baseline levels), but not late. In this “late-stimulus” condition, $\mu_{k}$ is set to 0 for the first pulse (*i* = 1), and reaches its expected value ($\mu_{k}$) by pulse 7. The transition from 0 at pulse 1 to $\mu_{k}$ at pulse 7 is governed by a logistic function with parameters chosen to result in a smooth transition between the first 3 and last 3 pulses (midpoint = 4, slope = 0.3). Although $\mu_{k}$ is near zero for the early pulses, σ is unchanged such that although the expectation for motion on pulse one is zero, the motion strength and direction will vary from trial to trial (see example trials in Fig. 2*B*). In other words, random draws of $X_{i}$ from distribution $N\left(\mu_{k},\sigma \right)$ where $\mu_{k}$ = 0 still carry motion information, albeit less correlated with the net motion outcome of the trial as a whole. The opposite is done for the “early-stimulus” condition (Fig. 2*C*), in which the first pulses maintain mean motion strength equal to $\mu_{k}$, and later pulses have a mean near zero. This stimulus design ensures that pulse sequences drawn from the $\mu_{k}$ = 0 Gaussian (i.e. “zero-mean trials”) maintain a 0 mean throughout all 7 pulses, regardless of whether the stimulus condition is flat, early, or late. These trials were difficult because the motion strength and direction of each pulse is small and independent of the sequence, and the net motion summed to a small directional outcome. About one quarter of macaque sessions also contained frozen seed trials, in which an identical stimulus was displayed for 5% to 10% of trials. These trials summed to exactly zero and the subject was rewarded at random.

All subjects began the experiments with the flat-stimulus condition. After multiple sessions of stable psychophysical performance within a condition, the stimulus was changed to either the late- or early-stimulus conditions. Finally, after multiple sessions of stable psychophysical performance under the second condition, they began the third and final condition. Subjects were exposed to only one stimulus condition per session and were not informed of which stimulus condition they were viewing before or during any given session.

### Data analysis

Sessions with a minimum of 250 successfully completed trials were included in data analysis. Sessions were excluded from analysis if subject accuracy was lower than 85% for the strongest motion values (17/235 sessions for macaques, 0/52 for humans). Additionally, 30 macaque sessions were excluded from analysis for having psychophysical thresholds >2 median absolute deviations about the median. Overall, 188 and 52 sessions were included for macaques and humans, respectively, with median session lengths of 632 and 295 successfully completed trials, netting a total of 129,922 and 15,275 trials overall.

All analyses were performed in Matlab (Mathworks). Subject choices in the direction-discrimination task were analyzed with a maximum likelihood fit of a three-parameter logistic function (Wichmann and Hill 2001) assuming a Bernoulli distribution of binary choices, in which the probability of a rightward choice is *p* and leftward choice is *1 – p*, where *p* is given by

(1) $$p=\gamma +\left(1-2\gamma \right)\left(\frac{1}{1+e^{-\beta \left(x-\alpha \right)}}\right),$$

where *x* is the net motion strength value (*z*-scored over all sessions for each subject separately), α is the bias parameter (reflecting the midpoint of the function in units of motion strength), β is the slope (i.e., sensitivity, in units of log-odds per motion strength), and γ captures the lapse rate as the offset from the 0 and 1 bounds. Error estimates on the parameters were obtained from the square root of the diagonal of the inverse Hessian (2nd derivative matrix) of the negative log-likelihood.

The temporal weighting kernel (which we also refer to as “temporal weighting strategy” or “temporal weighting profile”) was computed using ridge regression via maximum likelihood. The log posterior of the psychophysical weights is given by

(2) $$L\left(w\right)=\sum_{i=1}^{N}\left[Y\left(i\right)w^{T}X\left(i\right)-\log\left(1+\exp\left(w^{T}X\left(i\right)\right)\right)\right]+\lambda {\left\|w\right\|}^{2},$$

where Y∈{0,1} is a vector of choice on every trial and X is a matrix of the seven pulses on each trial, augmented by a column of ones (to capture bias). λ was estimated using evidence optimization (Sahani and Linden, 2003). Psychophysical weights are normalized by the Euclidean norm of the vector of weights. The seven temporal weights assigned to the seven motion pulses, *w*, were computed by using all trials within a session. These include trials where $\mu_{k}$ was set to zero (i.e. “zero-mean trials”, where motion on a given pulse is temporally independent of all other pulses in the sequence) and trials where $\mu_{k}$ was set to a non-zero value (“signal trials”, where motion is correlated over pulses). Psychophysical reverse correlation is traditionally performed on noise trials exclusively, but logistic regression effectively whitens the stimulus covariance, such that we could include all trials and increase our statistical power, regardless of whether they have correlated temporal structure. We verified the whitening step by comparing the psychophysical kernel computed on all trials to the kernel computed on only zero-mean trials and calculating the Pearson correlation between the pair of kernels (i.e., between the 7 weights of the all-trials-kernel and the 7 weights of the zero-mean-kernel) for each combination of subject and stimulus condition. This yielded 14 Pearson correlation values with a median of 0.886 ([0.819 to 0.952], 1 SEM) demonstrating a strong agreement between results of the two methods of reverse correlation for the subject-averaged data per condition. We also verified the whitening step at the level of individual sessions, using the same approach. This yielded 240 Pearson correlation values (one for each session) with a median of 0.846 ([0.829 to 0.864], 1 SEM), indicating a strong agreement between reverse correlation methods, even on single sessions.

The vector of weights, *w*, describes the temporal weighting adopted by the subject for a given set of trials. If the individual weights have a similar value, then that implies that the subject had weighted all pulses equally on average. If some weights are larger than others, that implies uneven weighting over time. We summarized temporal weighting by performing linear regression on the 7 weights and using the slope of the fit as a metric of temporal structure, where negative slopes indicate early psychophysical weighting and positive slopes indicate late. Comparisons of temporal weighting profiles across experimental conditions and species were assessed using the slope of the linear fit ± 95% confidence intervals. Wilcoxon sign tests were used to evaluate whether slopes differed significantly from zero. ANOVA was used to assess differences in mean slopes across experimental conditions. Bartlett’s test was used to evaluate differences in variance between distributions of slopes across experimental conditions. Table 1 details the statistical tests.

**Table 1.** Statistical tests.

Test use	Test	Data structure	Power
Psychophysical weighting calculated on all trials vs. only zero-mean trials	Pearson correlation	Linear	Subjects: median *r* = 0.886 [0.819 to 0.952], 1 SEM; single session: median *r* = 0.846, [0.829 to 0.864], 1 SEM
Differences in slope of linear fit to temporal weights between flat-, late-, and early-stimulus conditions in humans and macaques (Fig. 3)	Confidence intervals	Linear	Slope of linear fit, [95% confidence interval]; Macaques: flat: –0.050, [–0.069 to 0.031]; late: 0.051, [0.004 to 0.098]; early: –0.094, [–0.111 to –0.077]; Humans: flat: –0.013, [–0.032 to 0.006]; late: 0.053, [0.006 to 0.100]; early: –0.083, [–0.119 to –0.048]
Comparison of slope of linear fit to temporal weights during early-stimulus condition between humans and macaque subjects (Fig. 3)	Confidence intervals	Linear	Slope of linear fit, [95% confidence interval]; Humans: –0.013, [–0.032 to 0.006]; Macaques: –0.050, [–0.069 to –0.031]
Comparison of psychometric functions across conditions (Fig. 3)	Confidence intervals	Linear	Slope of psychometric function, [95% confidence interval]; Macaques: early: 3.39 [3.22 to 3.56], flat: 2.16 [2.13 to 2.18], late: 2.9339 [2.83 to 3.03]; Humans: early: 2.77 [2.56 to 2.99], flat: 2.14 [2.00 to 2.28], late: 2.60 [2.43 to 2.77]
Average slope of temporal weights for flat-, early-, and late-stimulus conditions compared to 0 (Fig. 5)	Wilcoxon sign test	Non-Gaussian	*p* < 0.0001, all conditions
Comparing group means for slopes of temporal weights for flat-, early-, and late-stimulus conditions (Fig. 5)	ANOVA	Gaussian	*p* < 0.0001
Comparing variance of slopes during flat stimulus condition vs. late- and early-stimuli (Fig. 5)	Bartlett’s test	Non-Gaussian	Flat-to-early, *p* < 0.0001; flat-to-late, *p* < 0.0001
Evaluating linear relationship between psychophysical threshold and slope of temporal weights (Fig. 5)	Pearson correlation	Linear	Flat: *r* = –0.29, *p* < 0.001; early: *r* = 0.46 *p* = 0.038; late: *r* = 0.05, *p* = 0.75
Evaluating linear relationship between psychophysical threshold and energy of temporal weights (Fig. 5)	Pearson correlation	Linear	Flat: *r* = 0.40, *p* < 0.0001; early: *r* = –0.004, *p* = 0.99; late: *r* = 0.31, *p* = 0.048

## Results

Overall, subjects performed more than 145,000 trials of a one-interval motion direction discrimination task. After viewing a sequence of motion pulses, they indicated the net perceived direction by moving their eyes to one of two targets (Fig. 1). In addition to the usual practice of varying the net strength and direction of motion across trials, the temporal statistics of the motion stimulus were manipulated within trials (in different series of sessions). Thus, sessions varied in whether the motion stimulus offered an equal amount of motion information over time (flat-stimulus condition) or whether some epochs contained more motion information than others (early-stimulus and late-stimulus conditions; Fig. 2*A–C*). This design is amenable to psychophysical reverse correlation such that in addition to computing standard subject performance as a function of stimulus strength, we calculated the psychophysical weights assigned by the subject to the motion stimulus over each epoch. We refer to the resulting weights as the temporal weighting strategy or temporal weighting profile. We found that both human and monkey observers shifted their temporal weighting profile in response to the differential temporal structure of motion statistics across the three stimulus conditions. We first present our subject-averaged results, followed by an examination of the differences between species and individual subjects.

### Temporal weighting strategies shift in response to stimulus statistics

Changes in temporal stimulus statistics led to clear shifts in the psychophysical weighting strategy in all subjects. We consider the flat-stimulus condition as a baseline, both because of the stationary statistics of the stimulus over time, and because the vast majority of stimuli used in the study of perceptual decision-making have temporally stationary statistics. In the flat-stimulus condition, subjects exhibited an inclination toward early weighting, with the highest weight on the first three pulses and then a steady decrease as time went on (Fig. 2*D*). The temporal weighting measurements were complimented by a standard analysis of subject psychometric performance. These indicate that observers were well engaged in the task and based their choices on the net strength and direction of the motion stimulus (Fig. 2*G*).

During late-stimulus sessions, subjects shifted their strategy to place higher weight on the later pulses, which more often carried high motion information and were therefore more reliably correlated with the final trial outcome. Temporal weights in the late-stimulus condition started low, increasing to a peak at the fifth or sixth motion pulse, followed by a decreased weight on the seventh (final) pulse (Fig. 2*E*). Although the late-stimulus condition had less motion information in early pulses, and consequently, less motion information overall compared to the flat-stimulus condition, subjects still exhibited standard psychometric performance, basing their choices on the net motion strength and direction (Fig. 2*H*).

In sharp contrast to the late-stimulus sessions, during early-stimulus sessions, subjects showed steep early weighting, where the first three pulses were weighted the highest followed by a large decrease (Fig. 2*F*). As with the late-stimulus condition, although the temporal weighting profile shifted markedly, both species exhibited standard psychometric performance (Fig. 2*I*).

**Figure 3.**
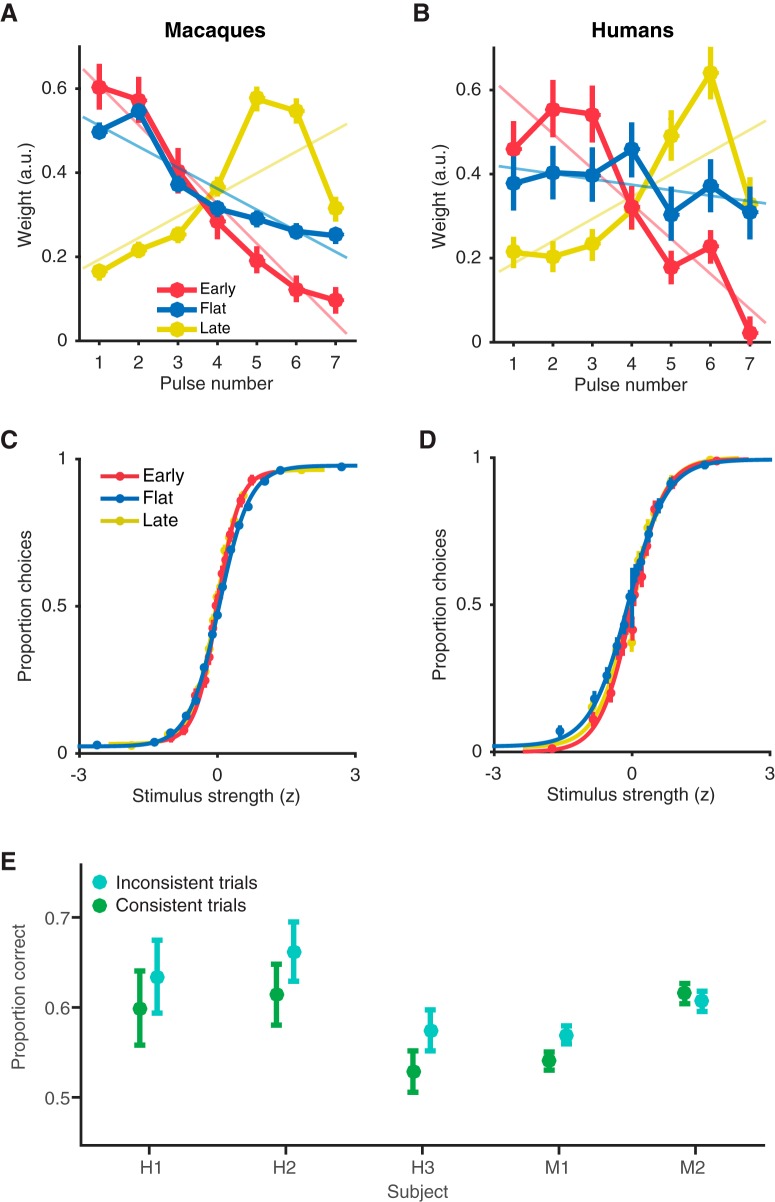
Comparison of temporal weighting and psychometric functions within species across stimulus conditions. ***A***, ***B***, Temporal weighting profiles for macaques (***A***) and humans (***B***) averaged over all sessions in the early-, flat-, and late-stimulus conditions, fitted by a linear model (semitransparent lines) to capture the overall trend of the weights. Error bars represent ± 1 SEM. ***C***, ***D***, Psychometric behavior of macaques (***C***) and humans (***D***) averaged over all sessions in the early-, flat-, and late-stimulus conditions, fitted by a logistic function to capture the dependence of choice on stimulus strength. Error bars represent ± 1 SEM. ***E***, Each subject’s proportion correct for inconsistent trials (where the strongest pulse is in the opposite direction of the full-trial, net direction) and difficulty-matched consistent trials (where the strongest pulse is in the same direction as the full-trial, net direction). Error bars represent 95% binomial confidence intervals.

The differences in temporal weighting strategies as a function of stimulus condition were robust and consistent across species (Fig. 3). Temporal weighting in the late-stimulus condition was significantly different from the weighting in the baseline flat-stimulus condition in macaques (Fig. 3*A*, flat: –0.050 [–0.069 to –0.031]; late: 0.051 [0.004 to 0.098]; slope of linear fit [95% confidence intervals]) and in humans (Fig. 3*B*, flat: –0.013 [–0.032 to 0.006]; late: 0.053 [0.006 to 0.100]). Temporal weighting in the early-stimulus condition was also significantly different from the weighting in the flat-stimulus condition for humans (Fig. 3*B*, flat: –0.013 [–0.032 to 0.006]; early: –0.083 [–0.119 to –0.048]), and in the monkey who performed the early-stimulus condition, M1 (Fig. 3*A*, flat –0.050 [–0.069 to –0.031]; early: –0.094 [–0.111 to –0.077]), although M1’s weighting strategy for the flat-stimulus condition was very early to begin with. Such early weighting for a flat-stimulus condition has been observed in various forms in previous reports (Huk and Shadlen, 2005; Kiani et al., 2008; Nienborg and Cumming, 2009; Katz et al., 2016; Yates et al., 2017; Odoemene et al., 2017). The difference in temporal weighting between the early-and late-stimulus conditions was highly significant in both species (humans, early: –0.083 [–0.119 to –0.048]. late: 0.053 [0.006 to 0.100]; macaques, early: –0.094 [–0.111 to –0.077]; late: 0.051 [0.004 to 0.098]).

In addition, no differences in temporal weighting strategy were observed between species within either the early- or late-stimulus conditions. In the flat-stimulus condition, in contrast, macaques exhibited an early weighting that was substantially steeper than that exhibited by the human observers (Fig. 3*A*, *B*, blue curve; humans: –0.013 [–0.032 to 0.006]; macaques –0.050 [–0.069 to –0.031]).

Lastly, the species-averaged psychometric functions exhibit a standard sigmoidal relationship between motion strength and choices in all stimulus conditions, demonstrating that subjects were properly engaged in the task. In the flat-stimulus condition, however, psychophysical performance was slightly decreased relative to performance in the early- and late-stimulus conditions, in both macaques (Fig. 3*C*; early: 3.39 [3.22 to 3.56], flat: 2.16 [2.13 to 2.18], late: 2.93 [2.83 to 3.03]) and humans (Fig. 3*D*; early: 2.77 [2.56 to 2.99], flat: 2.14 [2.00 to 2.28], late: 2.60 [2.43 to 2.77]).

In summary, observers performing perceptual decisions shifted their temporal weighting strategy dynamically and placed the most value on pulses with the highest motion expectation, whenever they were located in time.

### Ruling out extrema detection as a behavioral strategy

In all experiments, every trial was rewarded based on the true net direction of motion presented across the seven pulses, regardless of the underlying, generating distribution. Thus, integration of the motion information over all pulses would be ideal to maximize accuracy and reward. However, the possibility exists that subjects were not performing conventional temporal integration. For example, subjects could base their decisions on the strongest motion pulse within a trial as opposed to incorporating information from all pulses. Our stimulus design enabled us to perform a *post hoc* analysis to test whether subjects were performing this strategy of extrema detection (Fig. 3*E*).

We selected trials in which the direction of the strongest motion pulse (i.e., the pulse with the largest number of signal-carrying Gabor elements) was in conflict with the net direction of motion of the full trial (termed “inconsistent trials”). Most choices in these trials were in favor of the net direction of motion, as opposed to the direction of the extreme single pulse, in both human and macaque subjects (Fig. 3*E*). We then compared these inconsistent trials to trials that were matched for difficulty but in which the direction of the strongest pulse was in the same direction as the trial’s net direction (termed “consistent trials”). If subjects were performing extrema detection, then performance should be worse on inconsistent trials (where the strongest pulse was in the opposite direction of the net) compared to consistent trials. In contrast to this idea, no subject performed significantly worse on inconsistent trials, demonstrating that extreme pulse strengths did not influence subject choices nonlinearly in their favor, ruling the extrema detection strategy as unlikely in this task.

### Variability in temporal weighting strategy depends on stimulus condition

When averaged across sessions and subjects, temporal weighting profiles tell a fairly straightforward story: subjects adopt a late weighting strategy for the late-stimulus, an early weighting strategy for the early-stimulus, and a flat-to-early weighting strategy for the flat-stimulus. Here we sought to quantify the weighing strategy at a higher resolution by looking at performance for individual subjects and sessions.

**Figure 4.**
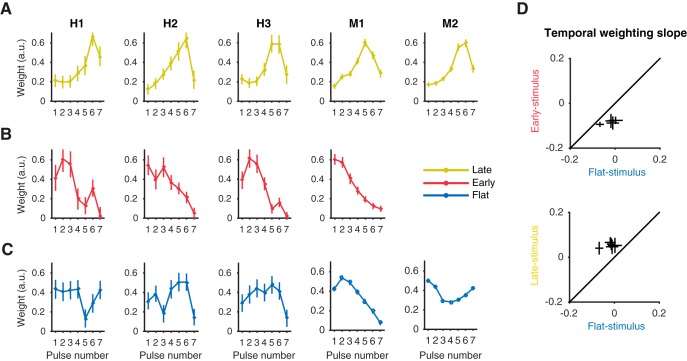
Temporal weighting strategies for individual subjects across stimulus conditions. ***A–C***, Average temporal weighting strategies for individual human and macaque subjects (columns) during the late-stimulus (***A***), early-stimulus (***B***) and flat-stimulus (***C***) condition. Error bars represent ± 1 SEM. ***D***, A within-subject comparison of the shift in temporal weighting strategies from flat-stimulus to early (top) and flat-stimulus to late (bottom), represented as the slope of the linear model fit to subject temporal weights. Error bars represent ± 1 SEM.

When each subject is considered individually, results were largely consistent with the average weighting profiles reported above. In the late-stimulus condition, human and macaque subjects’ weighting was extremely similar (Fig. 4*A*). All observers exhibited a single-humped psychophysical weighting profile in which peak weight was at pulse five or six, before a dropoff on pulse seven. Even the unexpected drop in weighting of the last pulse was shared. In the early-stimulus condition (Fig. 4*B*), subject M1 and subject H2 exhibited fairly linear early weighting patterns, and the remaining two human subjects showed slightly higher weights on the second pulses rather than the first, though still globally consistent with early weighting. Individual performance in the flat-stimulus condition (Fig. 4*C*), however, was more variable than in the late and early conditions. In monkey subjects, M1 showed very strong early weighting, while M2 exhibited U-shaped weights. Human subjects deployed generally flat weights on average but did so in idiosyncratic ways compared to the very stereotyped strategies of the early and late conditions. On average, each subject changed their temporal weighting as dictated by early- and late-stimulus conditions compared to the flat-stimulus condition (Fig. 4*D*). Overall, temporal weighing strategies adopted in the flat-stimulus condition were more variable than those adopted in the early- or late-stimulus conditions at the level of individual subjects.

**Figure 5.**
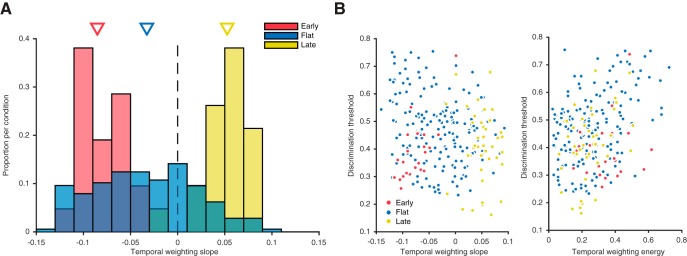
Variability in temporal weighting profiles and psychometric performance. ***A***, Distribution of temporal weighting profiles over sessions and subjects across the early-, flat-, and late-stimulus conditions, represented as the slope of the linear model fit to the temporal weights of each session. Negative slope values indicate an early weighting strategy; positive values indicate late. Triangles denote the median. ***B***, The relationship between psychometric performance (75% psychophysical threshold) and temporal weighting (slope of linear fit to temporal weights), over all sessions across the three stimulus conditions. ***C***, The relationship between psychometric performance (75% psychophysical threshold) and temporal weighting energy (sum of squared errors of temporal weight values from their mean), over all sessions across the three stimulus conditions.

When each session is considered individually, variability in temporal weighting strategy is evident both between and within each of three stimulus conditions. To quantify the degree of early versus late single-session weighting, we fitted a line to the seven temporal weights of the observer for each session and used the slope of this fit to summarize the temporal weighting profile: a positive slope indicates late weighting, a negative slope indicates early weighting, and a slope around zero indicates flat (or equal) weighting over time. The distribution of weighting slopes for all experimental sessions in the early-stimulus condition had an average of –0.079 (significantly less than zero, Wilcoxon sign test, *p* < 0.0001), with no single individual sessions having a slope greater than zero (Fig. 5*A*). The average slope for all late-stimulus sessions was 0.051 (significantly greater than zero, Wilcoxon sign test, *p* < 0.0001), with only 2 of 42 sessions having a slope less than zero. These distributions of weighting slopes reveal distinct populations across conditions (ANOVA, *p* < 0.0001), indicating that even at the resolution of single sessions, distinct strategies were adopted during the early- and late-stimulus conditions. The distribution of weighting slopes from the flat-stimulus condition had a mean of –0.0356, denoting slight early weighting (significantly less than zero, Wilcoxon sign test, *p* < 0.0001), but also differed in that it had a considerably larger range of results. The standard deviation of flat-stimulus weighting slopes was more than double that of the early- or late-stimulus weighting slope distributions (Bartlett’s test, flat-to-early, *p* < 0.0001; flat-to-late, *p* < 0.0001), indicating that subjects adopted a larger variety of temporal weighting strategies in this condition. It is worth noting that some of the variance in all three of the distributions comes from noise inherent to fitting a two-parameter linear model to the seven weights that constitute the temporal weighting strategy; nevertheless, the difference in distribution widths is substantial and therefore likely meaningful.

### Relationship between temporal weighting and psychometric performance

We next sought to examine the relationship between temporal weighting strategies and psychometric performance in the direction discrimination task. We compared the slope of the temporal weights to psychophysical threshold (i.e., the motion strength at which subject performed at 75% correct) for each stimulus condition (Fig. 5*B*). During the flat-stimulus condition, a negative correlation was present (*r* = –0.29, *p* < 0.001), indicating that adopting an early weighting strategy is detrimental to psychophysical performance. The early-stimulus sessions exhibited a positive correlation between temporal weighting slope and psychophysical threshold (*r* = 0.46, *p* = 0.038), indicating that in the early-stimulus condition, an early weighting strategy is preferable. Little to no correlation was observed in the late-stimulus sessions (*r* = 0.05, *p* = 0.75).

Perhaps more compelling was the relationship between psychophysical threshold and the energy of the temporal weights, where energy was measured as the sum of the squared residuals of each weight from the mean of the seven weights (Fig. 5*C*). This measurement gives us an estimation of variation or deviation from a consistent, flat weighting scheme. Here, flat-stimulus sessions showed a strong positive relationship between threshold and weighting energy (*r* = 0.40, *p* < 0.0001), demonstrating that during flat-stimulus sessions, employing weights that are highly variable from temporal uniformity (i.e., have high energy) is detrimental to psychophysical performance. Late-stimulus sessions showed a moderate positive correlation (*r* = 0.31, *p* = 0.048), and early-stimulus sessions showed no obvious linear relationship (*r* = –0.004, *p* = 0.99).

Taken together, larger variability in weighting and higher energy appear to be detrimental toward psychometric performance. These were most pronounced in the flat-stimulus condition, offering a potential explanation for the slight and unexpected decrease in psychophysical behavior during the flat-stimulus relative to early- and late-stimulus conditions (Fig. 3*C*, *D*).

## Discussion

We used psychophysical reverse correlation in the context of manipulations of temporal stimulus statistics to examine observers’ ability to update their temporal weighting strategy to match the time course of available evidence in a dynamic motion discrimination task. First, we found that when motion strength was systematically varied over time within a stimulus presentation, subjects changed their temporal weighting strategy to weight the periods of strong motion more heavily than those with weak motion. Second, weighting strategies were rather consistent across species and subjects, with the exception of the flat-stimulus condition. Third, session-to-session variability in strategy was greater in the flat-stimulus condition than in the late- and early-stimulus conditions. Each of these findings is discussed in more detail below.

### Temporal weighting likely reflects a combination of dynamic sensory reweighting and decision-making mechanisms

The observation of early sensory evidence exerting a larger effect on decisions than late evidence (i.e., early weighting) has been identified in prior work and has been interpreted within the context of a drift diffusion decision-making model. Early weighting is often interpreted as a straightforward consequence of accumulation to a decision bound—sensory data arriving after the bound has been hit does not impact the accumulator (Huk and Shadlen, 2005; Kiani et al., 2008; Okazawa et al., 2018; Kawaguchi et al., 2018). Just as past work has taken such early weighting as a signature of bounded accumulation, late weighting has been posited to reflect leaky integration. However, such models have been increasingly updated to accommodate either sort of behavioral signature (Usher and McClelland, 2001; Tsetsos et al., 2012; Bronfman et al., 2016). Thus, while time varying weighting has been identified before, it is almost always discussed as diagnostic about the structure of a decision-making mechanism, i.e., perfect or leaky integration to a bound (fixed or collapsing).

The shifts we identified in temporal weighting strategies show that time-varying weighting of a stimulus is a flexible strategy that adapts to the statistical structure of the stimulus. This flexibility highlights the possibility of a more direct reweighting of the sensory signal itself, regardless of downstream impacts, such as a bound or a leak in the sensory integration system. Temporal weighting strategies need not be solely the result of static decision-making mechanisms, but rather could reflect a dynamic strategy for directly weighting incoming stimulus. Another group made a similar observation (Cheadle et al., 2014), but in contrast to our findings, their results highlighted sequential dependencies within single trials and were interpreted via an appeal to normalization. Such normalization of evidence could be a part of many decision mechanisms, while the strategic shifts we identified here point to the possibility of a more general and flexible mechanism of dynamic reweighting of sensory evidence. By demonstrating an adaptive weighting strategy that easily shifts toward the most reliable motion information, we suggest that temporal weighting strategies could be interpreted as a gain on the incoming stimulus, rather then byproducts of mechanisms beyond the sensory stage of processing. Indeed, even when presenting a temporally uniform (flat) stimulus, the neural representation of that stimulus will impose its own time-varying signal-to-noise properties on whatever downstream circuits may receive that information for integration or other such computations (Osborne et al., 2004; Churchland et al., 2010; Yates et al., 2017). It is therefore possible that changes in temporal weighting strategy in the presence of temporally dynamic stimuli are due to direct reweighting of the time-varied responses in sensory circuits.

It remains to be seen whether the observed time-varying weighting in sensory brain areas can be changed in response to temporal manipulations of the stimulus of the sort we employed, but the well documented effects of temporal attention in multiple visual cortical areas (Ghose and Maunsell, 2002) lend credence to this hypothesis. Likewise, changes in spike-count correlation structure with task instruction have been shown to reflect feedback in early sensory areas (Bondy et al., 2018), suggesting a possible source for context-dependent reweighting in the current experiments as well. Notably, our data do not rule out the impacts of decision mechanisms. The existence of a bound at later stages of decision formation could still interact with stimulus reweighting. This could be further sculpted by urgency signals or time-varying bounds (Ditterich, 2006; Bogacz et al., 2006; Churchland et al., 2008; Cisek et al., 2009; Hanks et al., 2014; Okazawa et al., 2018). In fact, a potential example of such an interaction between stimulus reweighting and a bounded decision mechanism might be present in the late weighting behavior we observed, which often manifested with a seemingly idiosyncratic, low weight on the final pulse. Although subjects clearly down-weighted the first few pulses, and up-weighted pulses 5 and 6, the low weight on the final pulse could be explained as a byproduct of achieving the bound before the end of the stimulus, even in the late-stimulus condition.

### Increased variability during the flat-stimulus condition provides insights into previous variability in the literature

Variability in temporal weighting strategy during the flat-stimulus condition was far larger than in either the early- or late-stimulus conditions. This substantial variability is of general relevance to the study of evidence accumulation, because it is typically performed using stimuli that are similar to our flat-stimulus condition, in that their expectation is stationary over time. Although the average weighting strategies for both humans and macaques in the flat-stimulus condition trend toward early weighting, session-by-session analysis of weighting slopes revealed robust variability (Fig. 5). Few if any prior studies have characterized individual session strategies, likely owing to low statistical power of alternate designs that rely on *post hoc* characterization or infrequent probe trials. Our results suggest that even individual subject averages may gloss over strategic variability within the observer that occurs over sessions. Likewise, even the relatively high-resolution session averages we present here may mask variability over single trials, variability that current trial-based psychophysical methods lack the resolution to resolve. Consequently, all temporal weighting strategies presented here (and elsewhere, as far as we know) are computed as an average over multiple trials, each with a potentially unique temporal weighting strategy.

The large session-by-session variability in weighing strategies observed here may serve to reconcile those presented elsewhere. In the flat-stimulus condition, all time points (i.e., pulses) are equally informative of the trial outcome, and thus the flat-stimulus condition is more forgiving of different temporally biased weighting strategies compared to the early and late conditions, for which only approximately half of the stimulus contained informative evidence on average. Thus, increased variability in weighting strategies during the flat-stimulus condition compared to early- and late-stimulus conditions is likely a consequence of temporally uniform stimulus statistics—a feature of most evidence accumulation studies.

The consistency of temporal weighting across species displayed in the late and early stimulus conditions also suggests that, at least for humans and macaques, interspecies differences need not be a major player in variability of weighting. This is of possible broader interest, for example, in linking to rodent work (Erlich et al., 2015, Scott et al., 2015, Morcos and Harvey 2016, Pinto et al., 2017, Odoemene et al., 2017, Licata et al., 2017).

One discrepancy across species was present in the flat-stimulus condition, in which macaque subjects (on average, but most pronounced in M1) displayed an early-weighting strategy (despite flat stimulus expectation) compared to the flat-weighting strategy displayed by humans. This could be for a number of reasons. Macaques performed many more trials and sessions than human subjects, raising the possibility that extensive training may result in faster decisions, based on early epochs of the stimulus. This may be further accentuated by a desire to perform more trials and obtain more liquid reward (a factor not included in experiments with human subjects). While such a strategy does not in fact change the trial duration or, in turn, the speed-accuracy trade-off, it might factor into macaques’ behavior. It is noteworthy that the species difference is present only in the flat-stimulus condition, and not the time-variant conditions. We believe this is because the flat-expectation and fixed-duration design is lenient with respect to temporal weighting, granting subjects the liberty to adopt any number of temporal weighting strategies (Fig. 5). This is very different from the time-varying conditions, which place clear constraints on the temporal weighting strategies that would benefit the subject. These considerations may serve to reconcile past conflicting results in different task designs and species and inform new work going forward.

### Difficulties in interpreting temporal weighting strategies in light of stimulus and task design

Stimulus and task design must be considered to properly interpret the shape of a temporal weighting strategy. Given that single trials are always rewarded based on the true net motion presented, regardless of their underlying distribution, all motion pulses are always informative. Therefore, it is intuitive that highest overall accuracy would be realized via a strategy that assigns equal weighting across all pulses. However, this was not uniformly present in our dataset, indicating that subjects did not perform the task optimally. Importantly, the assumption of equal weighting is only one part of an optimality argument, as equal but low weighting of incoming sensory data would of course be suboptimal too. Complete optimality of the decision mechanism is a difficult standard to assess without a detailed characterization of signal and noise in both the stimulus and the sensory neural representation (Geisler, 1989). Given that most relevant experimental paradigms do not avail themselves straightforwardly to a formal and complete ideal observer model, the shape of the temporal weighting provides only partial insight into decision formation, without a gold standard for the overall level of accuracy.

A similar difficulty is present in evaluating the optimality of temporal integration in fixed-duration tasks. Classically, tests of optimal temporal integration appeal to the relation between viewing duration and accuracy (Kiani et al., 2008, Katz et al., 2015). However, two issues we have discussed with respect to temporal weighting also speak to limitations in evaluating optimality in temporal integration via the relation between viewing duration and accuracy. First, underweighting the sensory evidence before accumulating is suboptimal but is not captured by such an analysis, which would lump such an effect in with sensory noise. Second, although a sensory stimulus may have certain temporal properties, the neural representation of the sensory stimulus is likely to have time-varying signal-to-noise properties (Osborne et al., 2004; Churchland et al., 2010; Yates et al., 2017). Standard viewing-duration analyses do not distinguish between the stimulus and the neural signals that are actually used. These two issues likely interact, with the potential for dynamic strategic weighting to either mirror or compensate for the dynamics of the incoming sensory stream—making canonical functional forms of the relations between accuracy and duration rather imperfect tests of a unique posited mechanism (Huk et al., 2017).

Other aspects of experimental design may increase the complexity of inferences drawn from the assessment of temporal weighting as well. For example, although early weighting may be a general default state (potentially driven by extensive training and/or the default structure of decision mechanisms), variable duration paradigms may fortify an early weighting strategy. Variable duration paradigms can be thought of as loosely analogous to our early-stimulus condition, in that as time progresses, the expected stimulus strength falls off (owing to the end of the variable-duration stimulus). Reaction time tasks can also facilitate an early weighting strategy, as the subject is typically incentivized to respond as fast as possible, placing more weight on early samples within a stream (Okazawa et al., 2018). Lastly, time-varying confidence may play a role in shaping temporal weighting strategies too (Kiani and Shadlen, 2009; Kawaguchi et al., 2018). Taken together, the patterns of selective temporal weighting we have discussed imply that it will be fruitful to characterize evidence accumulation at a fine grain and to allow for the potential interplay of both flexible and fixed mechanisms in sculpting the resulting dynamics.

Our characterizations of temporal weighting are of course inherently limited by the assumptions of logistic regression. While it is clear that subjects weigh temporal sections of the stimulus in proportion to their expected motion signal, it seems unlikely that the way the brain performs this task is completely described by logistic regression. There are likely a cascade of nonlinearities between stimulus and response that cannot be fully described by a set of linear weights passed through a sigmoid, which implies that the exact pattern and magnitudes of an individual temporal kernel are an incomplete description of the decision process. However, given the close correspondence between kernels computed using only flat-expectation, zero-mean (noise) trials and kernels computed using all trials (where there is often temporal correlation in the stimulus), any nonlinearity in mapping from stimulus to sensory evidence appears to have a minimal impact on our core result: differences between temporal stimulus statistics can exert systematic and interpretable effects on temporal weighting strategies.

More generally, our results provide an opportunity to reconnect perceptual decision-making models with other frameworks for information integration. For example, the dynamic temporal weighting we observed has a direct connection to classical Bayesian integration (Hillis et al., 2004; Körding and Wolpert, 2006; Knill 2007; Angelaki et al., 2009; Fetsch et al., 2009). Over repeated exposure to a given stimulus condition, subjects learn to weigh stimulus cues according to reliability. In our experiment, time epochs (motion pulses) can be thought of as akin to cues: each motion pulse is a cue toward the trial’s net direction, but during early- and late-stimulus conditions subjects must learn to down-weight noisy epochs and up-weight reliable ones. Cue combination with reliability-based weighting has been commonly observed both within and across sensory domains (Hillis et al., 2004; Morgan et al., 2008; Angelaki et al., 2009; Fetsch et al., 2009, 2011). While Bayesian integration has been discussed specifically with respect to bounded accumulation (Beck et al., 2008), it also lends itself to a reliability-based readout of a temporally dynamic sensory representation. Time points in the sensory response with a higher signal-to-noise ratio may be more strongly weighted toward choice. For example, as discussed above, a tendency toward early weighting in the flat stimulus condition could be reflective of temporal variation during sensory encoding rather than an effect of downstream mechanisms such as a bound. We are encouraged by this mapping to a Bayesian framework and the implication that further manipulations of reliability of evidence in time can continue to build tighter links (or reveal contrasts) between cue integration and temporal integration (Katz et al., 2015; Hanks et al., 2011).

In summary, past work has used reverse correlation and time-varied stimuli to probe temporal integration. In the present study, we used a reverse correlation task in the context of tractable manipulations of stimulus statistics, allowing for direct control over a subject’s temporal weighting strategy. Although the neural correlates of such changes remain uncertain, the ability to both manipulate and characterize temporal weighting strategies should provide a powerful tool for neurophysiological experiments to come.

## Synthesis

Reviewing Editor: Li Li, New York University Shanghai Decisions are customarily a result of the Reviewing Editor and the peer reviewers coming together and discussing their recommendations until a consensus is reached. When revisions are invited, a fact-based synthesis statement explaining their decision and outlining what is needed to prepare a revision will be listed below. The following reviewer(s) agreed to reveal their identity: NONE.

Both reviewers believe the ms addressed an important question on perceptual decision making in both humans and monkeys. Both reviewers also raised several important concerns that should be addressed before the ms can be considered for publication. I list their concerns below.

Reviewer 1

I thought the paper is carefully written and the changes in the psychophysical kernels across conditions look promising. However I have a few concerns about analysis and interpretation as explained below.

1. One concern is that the authors used all trials to compute the psychophysical kernel. Although their logistic regression takes into account the mean stimulus level, it is recommended to use only zero-mean trials for reverse correlation because any potential nonlinearity in mapping of sensory input to evidence can distort the net effect of mean stimulus levels. Suppose, for example, that subjects are relatively insensitive to small differences in proportions of left/right patches but very sensitive to large differences (relaxed version of extrema detection strategy; note that although the authors claimed that the extrema detection strategy does not explain 60% of trials, the remaining 40% of trials may still be affected by that (p.12 l.208-)). Under this assumption, the changes in psychophysical kernel across conditions will be mostly explained by the temporal profile of stimuli itself because different temporal profiles have different probabilities of showing extreme proportions. Thus, I would like to confirm that the difference in kernels across conditions persists even when the authors used only the zero-mean trials, whose stimulus profiles are identical across conditions.

2. It was unclear how the authors interpret the lack of change in performances across conditions. The flat-stimulus condition clearly had more sensory evidence, but the subjects did not show any improvement of performance (Fig. 3C-D). Furthermore, the scatter plot of psychometric thresholds (Fig. 5B-C) appears to indicate that subjects showed poor behavioral thresholds (&gt;0.6) in many sessions for the flat-stimulus condition, while those poor performances were not seen in the other conditions. Even if the sensory weights happened to be more variable in the flat-stimulus condition, it does not readily explain these poor performances. Could it be because of the lack of subjects' expertise or engagement during the flat-stimulus condition? Were sessions of different conditions interleaved during the experiment, or is there any bias in the way each condition was introduce? For example, if the flat-stimulus was mostly used in the earlier sessions when subjects weren't fully trained, the results shouldn't be compared with other conditions.

3. The authors discuss the implications for stimulus and task design (p.32-33). I agree with many of the authors' arguments about potential pitfalls of several task designs (although some of them may not be particularly relevant to the present results), but I think that the variability of kernels in this study (Fig. 5A) specifically points to a difficulty in interpreting the data when one uses fixed stimulus duration. Perhaps a fair conclusion would be that one should be always aware of the downsides of any task designs including the fixed duration task.

Minor

1. Is equation 2 correct? Shouldn't it be L(w) = Sigma[YwX “-”(minus, not plus) log(1 + exp(wX))] + lambda |w|2 as it is a logarithm of logistic function?

2. In Fig 2A, labeling the zero-mean trials as “noise” may be confusing, as the noise has different meaning (counter-phase flicker elements) in this task (the same condition is labeled “zero” in Fig. 1C.)

Reviewer 2

The experiments are well executed and presented. But there are a couple of lacunae in the analyses which need to be addressed to make the results more interpretable. Further there are some important differences between macaque and human subjects that need clarification and discussion.

Major issues:

1. There is very little comparison between the psychometric functions in the three stimulus conditions presented in the manuscript. Such a comparison can provide different insights into the results that temporal weighting functions cannot.

For e.g., if the subjects are using relevant information throughout the trial in each of the stimulus conditions, then the slope and threshold of the psychometric functions should be comparable across the three conditions for a given amount of total evidence. However, visual examination of the psychometric functions in Fig. 3 seem to suggest that, paradoxically, both human and especially macaque subjects seem to do better when the signal is restricted to a subset of the trial (either early or late). If this is true, then it could be that on any given trial in the flat condition, they are using information from a small subset of stimulus epochs. While this does not detract from the authors' conclusion that the subjects can switch which epochs of a trial the subjects use, it does bear on the complexity of the switching process.

2. Following on from the above comment, a particular extreme strategy could be that the subjects use only the most salient single epoch to base their decisions. The authors perform an analysis to rule this out, but surprisingly, this is confined to the methods section (lines 203-212) and is short on details. This is an analysis and so, would belong in results. Also, can the authors elaborate on this? Why would the subjects choose the extreme strong pulse side on ~40% of the trials even when the net motion is going against it? What are the results if this analysis is extended to trials where the two largest pulses go against the net motion direction?

3. There is a significant difference between the temporal weighting profiles used by the macaques and humans in the flat stimulus condition. The monkeys' weighting profile in the flat condition looks very similar to their profile in the early-stimulus condition (comparing blue and red lines in Fig. 3A). While the authors do acknowledge the differences in results, they are papered over in discussion. For eg.,

* Line 453: “...weighting strategies were rather consistent across species...“ is not completely correct

* Lines 578 - 584: “ ... variable duration .... Reaction time task .... can facilitate early weighting strategy ...” This seems to be applicable only to humans as the fixed duration task used in the current study still results in early weighting strategy in macaques.

These species differences could be clarified better in discussion.

Minor issues:

1. It is not clear from the methods that the different temporal profile stimuli were used in different sessions. More details regarding how the sessions were ordered is warranted in the methods section.

2. The results section starting from line 422 could be hived off into its own section. It does not really fit under the heading of the section it currently is in.

## Author Response

Reply to reviewers and editor

We thank the editor and reviewers for their constructive comments and especially for pointing out the need for clarification of some key analyses.

The reviewers raised three main concerns: (i) that our reverse correlation analysis might result in different outcome if performed on zero-mean trials exclusively (rather than all trials), (ii) that the subjects in this study might be making decisions by detecting strong motion pulses (i.e. extrema detection) rather than integrating, and (iii) that a discussion surrounding psychometric functions across stimulus conditions is lacking.

We have addressed all comments raised by the reviewers below, revised the main text and figures accordingly, and believe these changes have made the paper substantially stronger.

We have also noticed that our Introduction section was longer than that prescribed in the author guidelines. We have edited it to meet the word limit.

Please note that parenthetical callouts to specific line numbers correspond to “text-only” versions of the manuscript.

Reviewer 1

1). One concern is that the authors used all trials to compute the psychophysical kernel. Although their logistic regression takes into account the mean stimulus level, it is recommended to use only zero-mean trials for reverse correlation because any potential nonlinearity in mapping of sensory input to evidence can distort the net effect of mean stimulus levels. Suppose, for example, that subjects are relatively insensitive to small differences in proportions of left/right patches but very sensitive to large differences (relaxed version of extrema detection strategy; note that although the authors claimed that the extrema detection strategy does not explain 60% of trials, the remaining 40% of trials may still be affected by that (p.12 l.208-)). Under this assumption, the changes in psychophysical kernel across conditions will be mostly explained by the temporal profile of stimuli itself because different temporal profiles have different probabilities of showing extreme proportions. Thus, I would like to confirm that the difference in kernels across conditions persists even when the authors used only the zero-mean trials, whose stimulus profiles are identical across conditions.

The reviewer expressed a concern that our reverse correlation analysis might be affected by nonlinear mapping of stimulus to sensory evidence, a concern that is especially relevant to high-signal trials that differ across the early-, flat-, and late-stimulus conditions. We shared the reviewer's concerns about this issue regarding whether to include all trials or just the zero-mean trials. We therefore internally verified this both in simulation and on the experimental data. Results of the simulation are provided in this response only, while results from our data analysis are now included both in this response (in detail, along with figures) as well as in the revised manuscript (lines 246 -- 260).

Simulation results:

We performed a set of simulations to validate that reverse correlation coupled with whitening of the stimulus covariance matrix produces a similar result when using all trials vs. when using only zero-mean trials. In this exercise, we simulated choices by passing the actual stimuli presented to each subject through a temporal weighting kernel (either early, flat, or late, referred to as the “true kernel”), and then performed reverse correlation on either all trials, or only the zero-mean trials, in an attempt to recover the true kernel. In both cases, the estimated kernels matched the true kernels used to generate the choices and were similar to one another (Figure 1 of this response). This concordance demonstrates that reverse correlation produces a similar result regardless of whether all trials are used in the analysis, or only zero-mean trials.

Data analysis:

We also validated our approach on the data reported in the manuscript. We compared kernels computed by reverse correlation of all-trials vs. zero-mean trials from individual subjects in each condition. Results from the two analyses are qualitatively consistent in almost all cases (Figure 2 of this response). To summarize this quantitatively, we calculated the Pearson correlation between the 7 weights of the 'all-trials kernel' and the 7 weights of the 'zero-mean-trials kernel'. The median Pearson r was 0.886 ([0.819 0.952], 1 SEM), indicating strong agreement between reverse correlation methods. These results have been added to the revised manuscript (lines (lines 249 -- 256). One obvious inconsistency is present in H3 during the late-stimulus condition, where the zero-mean-trials kernel is flatter than the all-trials kernel. This and other mismatches may be due to the fact that, as the reviewer suggests, subject strategies are not necessarily logistic regression per se (i.e. a linear weight on each pulse, passed through a sigmoid). This may be causing slight variation between kernels computed on all-trials vs. those computed on zero-mean trials alone. However, given the close correspondence between the two methods of kernel measurement, and the validation of our method in simulation, any nonlinearity in mapping from stimulus to sensory evidence appears to have a minimal impact and does not change our results.

Next, we extended our quantitative analysis to the level of single sessions. We compared all-trials-kernels to noise-trials-kernels by calculating the Pearson correlation between each session kernel pair (Figure 3 of this response). Agreement between kernels is generally strong, with a median Pearson r of 0.846 ([0.829 0.864], 1 SEM). The results of this analysis are also present in the revised manuscript (lines 256-260).

Lastly, the reviewer also raises the issue of extrema detection, which warrants thorough consideration. We address this point in detail in response to Comment 2 from Reviewer 2.

2). It was unclear how the authors interpret the lack of change in performances across conditions. The flat-stimulus condition clearly had more sensory evidence, but the subjects did not show any improvement of performance (Fig. 3C-D). Furthermore, the scatter plot of psychometric thresholds (Fig. 5B-C) appears to indicate that subjects showed poor behavioral thresholds (&gt;0.6) in many sessions for the flat-stimulus condition, while those poor performances were not seen in the other conditions. Even if the sensory weights happened to be more variable in the flat-stimulus condition, it does not readily explain these poor performances. Could it be because of the lack of subjects' expertise or engagement during the flat-stimulus condition? Were sessions of different conditions interleaved during the experiment, or is there any bias in the way each condition was introduce? For example, if the flat-stimulus was mostly used in the earlier sessions when subjects weren't fully trained, the results shouldn't be compared with other conditions.

The reviewer points out that a number of sessions have poor psychophysical performance, and that these sessions are only in the flat-stimulus condition. This is a concern because psychophysical behavior should ideally be stable within a normal range of sensitivity when comparing temporal weighting strategies across the early-, flat- and late-stimulus conditions. To address this concern, we have made our exclusion criteria more stringent and removed sessions with high psychophysical thresholds from analyses in the manuscript (lines 212-219).

To elaborate, we identified “poor performance sessions” as sessions with psychophysical thresholds greater than the median + 2*median absolute deviation. 30 out of 270 sessions were flagged as poor performance and examined. We found that these sessions largely trend towards our exclusion criteria for lapse rates and number of trials. These sessions were all from the flat-stimulus condition, all performed by the macaque subjects, and appear to be outliers of the distribution. We did not detect that these were a consequence of partial training or interleaving conditions and cannot point to the exact source of poor performance (we note that there were many more flat sessions than the time-varying conditions, so these sessions may simply be pulls from the tails of the same distribution). To restrict our analyses to standard levels of psychophysical performance, we decided to exclude outlier sessions (i.e. sessions with thresholds larger than the median+2*median absolute deviation) from the manuscript to achieve a clean and faithful comparison of temporal weighting strategy across stimulus-conditions.

The reviewer also requests a more thorough interpretation of the psychophysical performance across stimulus conditions. Please see our response to comment 1 of Reviewer 2, where we address this topic in detail and note the changes made to the revised manuscript.

3). The authors discuss the implications for stimulus and task design (p.32-33). I agree with many of the authors' arguments about potential pitfalls of several task designs (although some of them may not be particularly relevant to the present results), but I think that the variability of kernels in this study (Fig. 5A) specifically points to a difficulty in interpreting the data when one uses fixed stimulus duration. Perhaps a fair conclusion would be that one should be always aware of the downsides of any task designs including the fixed duration task.

We appreciate the reviewer's thoughts regarding our discussion of task and stimulus design, and agree that our conclusions are primarily relevant to fixed duration stimuli. We have now added a number of lines discussing the potential pitfalls of the fixed duration task specifically, acknowledging its limitations in study at hand as well as more generally in the field of perceptual decision (lines 595-609).

Minor Issues

1. Is equation 2 correct? Shouldn't it be L(w) = Sigma[YwX “-”(minus, not plus) log(1 + exp(wX))] + lambda |w|2 as it is a logarithm of logistic function?

Thank you for catching that error. Yes, equation 2 (line 236) has been corrected.

2. In Fig 2A, labeling the zero-mean trials as “noise” may be confusing, as the noise has different meaning (counter-phase flicker elements) in this task (the same condition is labeled “zero” in Fig. 1C.)

Thank you for pointing this out. To avoid confusion, we have changed the labels in Figure 2A and Figure 1C to “zero-mean.”

Reviewer 2

1). There is very little comparison between the psychometric functions in the three stimulus conditions presented in the manuscript. Such a comparison can provide different insights into the results that temporal weighting functions cannot.

For e.g., if the subjects are using relevant information throughout the trial in each of the stimulus conditions, then the slope and threshold of the psychometric functions should be comparable across the three conditions for a given amount of total evidence. However, visual examination of the psychometric functions in Fig. 3 seem to suggest that, paradoxically, both human and especially macaque subjects seem to do better when the signal is restricted to a subset of the trial (either early or late). If this is true, then it could be that on any given trial in the flat condition, they are using information from a small subset of stimulus epochs. While this does not detract from the authors' conclusion that the subjects can switch which epochs of a trial the subjects use, it does bear on the complexity of the switching process.

We agree with the reviewer that it is important to evaluate the psychometric functions (PMF) across conditions. Specifically, the reviewer points out that PMFs for the flat-stimulus condition are, unintuitively, shallower than those of the early- and late-stimulus conditions in both humans and macaques. This issue has been partially addressed by the exclusion of poor performance sessions (see response to comment 2 from reviewer 1), but this does not address the reviewers' requests to more thoroughly compare psychometric performance across conditions. Thus, we have updated the manuscript to (a) report the differences in PMFs across species and conditions (lines 344-352), and (b) focus on the relationship between PMF and temporal weighting strategy (lines 431-457) in a new section of the Results titled “Relationship between temporal weighting and psychometric performance”, per recommendation of reviewer 2, minor comment 2. These changes are explained in detail below.

Figure 5 of the submitted manuscript provides insight towards the cause of increased psychometric thresholds during the flat-stimulus condition. The analyses in panels 5B and 5C demonstrate a significant correlation between psychophysical threshold and temporal weighting slope (Figure 5B), as well as weighting energy (Figure 5C), where energy is the sum of the squared residuals of each weight from the mean of the seven weights. The relationship is strongest by far for the flat-stimulus condition, where high weighting energy and more negative slopes correlate with higher (worse) PMF thresholds. This might be a consequence of the large variability in weighting strategies for the flat-stimulus condition, where subjects may assume idiosyncratic strategies that are detrimental to performance. This is one potential explanation to the decreased psychophysical performance of subjects in the flat-stimulus condition compared to the early and late. The manuscript text has been edited to elaborate on this relationship and to connect back to the psychometric functions in Figure 3 (lines 453-457).

2). Following on from the above comment, a particular extreme strategy could be that the subjects use only the most salient single epoch to base their decisions. The authors perform an analysis to rule this out, but surprisingly, this is confined to the methods section (lines 203-212) and is short on details. This is an analysis and so, would belong in results. Also, can the authors elaborate on this? Why would the subjects choose the extreme strong pulse side on ~40% of the trials even when the net motion is going against it? What are the results if this analysis is extended to trials where the two largest pulses go against the net motion direction?

We thank the reviewer for requesting clarification of the extrema detection analysis. We have now elaborated the analysis and updated the manuscript accordingly. The text describing this analysis has been moved from the Methods to a new section in the Results, titled “Ruling out extrema detection as a behavioral strategy” (lines 357-379). Furthermore, we have added a new panel to Figure 3 of the revised manuscript. Details of the expanded analysis are presented below.

The previous analysis evaluated performance on trials where the direction of the strongest pulse differed from the net direction (termed “inconsistent trials”). In these trials, subjects chose in favor of the extreme pulse ~40% of the time. The reviewer points out that although the majority of choices are not in favor of the extreme pulse, this number seems large. This number is not quantitatively interpretable by itself, however, because most inconsistent trials come from the zero-mean distribution, meaning that these trials were very difficult. Thus, it was unclear in the previous analysis whether choices on those 40% of trials were driven by an extrema detection strategy, or simply instances of just-above-chance performance expected for trials of this difficulty.

To address whether the low rates of percent correct on inconsistent trials were due to extrema detection or pure trial difficulty, we examined trials in which the net direction was the same as the strongest pulse for each subject (termed “consistent trials”). Critically, the consistent trials were difficulty-matched to the net coherence of the inconsistent trials. If, for example, subjects have higher accuracy on consistent trials than inconsistent trials, we could infer that the strongest pulse is nonlinearly informing subject decision, consistent with extrema detection. We found that no subjects performed better on the consistent vs. the inconsistent trials (Figure 4 of this response), leading us to reject the hypothesis that subjects are performing extrema detection.

We are very grateful to the reviewer for having motivated us to perform this elaborated analysis and decided to include this figure as a panel in Figure 3 of the manuscript.

3). There is a significant difference between the temporal weighting profiles used by the macaques and humans in the flat stimulus condition. The monkeys' weighting profile in the flat condition looks very similar to their profile in the early-stimulus condition (comparing blue and red lines in Fig. 3A). While the authors do acknowledge the differences in results, they are papered over in discussion. For eg.,

* Line 453: “...weighting strategies were rather consistent across species...” is not completely correct

* Lines 578 - 584: “ ... variable duration .... Reaction time task .... can facilitate early weighting strategy ...” This seems to be applicable only to humans as the fixed duration task used in the current study still results in early weighting strategy in macaques.

These species differences could be clarified better in discussion.

The reviewer is correct in pointing out the difference in temporal weighting profiles between humans and macaques in the flat-stimulus condition. We have clarified this point per reviewer request by expanding the Discussion text (lines 560-577).

A number of factors unique to macaques could lead to the heightened early weighting during the flat stimulus condition relative to humans. In the revised manuscript, we now discuss the potential impacts of overtraining, and reward incentive. While we find the difference intriguing, we can only speculate as to its cause. However, we believe it is especially important to highlight this species difference as part of a larger trend of increased variability in temporal weighting during flat-stimulus sessions compared to the early- and late-stimulus sessions. The flat-expectation and fixed-duration design in this study (and many others) is rather permissive, and potentially conducive to variable forms of temporal weighting and integration, which together with extensive training of the macaque subjects, may have led to a more pronounced early temporal weighting. We have added these considerations to the manuscript and noted that increased variability of temporal weighting during flat-expectation stimuli has important implications for reconciling past conflicting results and informing new work going forward.

Minor Issues

1. It is not clear from the methods that the different temporal profile stimuli were used in different sessions. More details regarding how the sessions were ordered is warranted in the methods section.

Thank you for pointing out that session order was unclear. We have clarified this ambiguity by adding text at the end of the “Temporal manipulation of stimulus” section in the Methods (lines 203-209).

2. The results section starting from line 422 could be hived off into its own section. It does not really fit under the heading of the section it currently is in.

Per the reviewer's suggestion, we have created a new section titled “Relationship between temporal weighting and psychometric performance” for this text and new text in response to comment 1 from reviewer 2 (lines 431-457).
